# Cannabis and tobacco smoke are not equally carcinogenic

**DOI:** 10.1186/1477-7517-2-21

**Published:** 2005-10-18

**Authors:** Robert Melamede

**Affiliations:** 1 Biology Department, 1420 Austin Bluffs Parkway, University of Colorado, Colorado Springs, 80918, USA; 2 Bioenergetics Institute, 1420 Austin Bluffs Parkway, University of Colorado, Colorado Springs, 80918, USA

**Keywords:** marijuana, tobacco, cancer, smoke, cannabinoids, carcinogens, nicotine

## Abstract

More people are using the cannabis plant as modern basic and clinical science reaffirms and extends its medicinal uses. Concomitantly, concern and opposition to smoked medicine has occurred, in part due to the known carcinogenic consequences of smoking tobacco. Are these reactions justified? While chemically very similar, there are fundamental differences in the pharmacological properties between cannabis and tobacco smoke. Cannabis smoke contains cannabinoids whereas tobacco smoke contains nicotine. Available scientific data, that examines the carcinogenic properties of inhaling smoke and its biological consequences, suggests reasons why tobacco smoke, but not cannabis smoke, may result in lung cancer.

## 

Tobacco has dramatic negative consequences for those who smoke it. In addition to its high addiction potential [1], tobacco is causally associated with over 400,000 deaths yearly in the United States, and has a significant negative effect on health in general [2]. More specifically, over 140,000 lung-related deaths in 2001 were attributed to tobacco smoke [3]. Comparable consequences would naturally be expected from cannabis smoking since the burning of plant material in the form of cigarettes generates a large variety of compounds that possess numerous biological activities [4].

While cannabis smoke has been implicated in respiratory dysfunction, including the conversion of respiratory cells to what appears to be a pre-cancerous state [5], it has not been causally linked with tobacco related cancers [6] such as lung, colon or rectal cancers. Recently, Hashibe et al [7] carried out an epidemiological analysis of marijuana smoking and cancer. A connection between marijuana smoking and lung or colorectal cancer was not observed. These conclusions are reinforced by the recent work of Tashkin and coworkers [8] who were unable to demonstrate a cannabis smoke and lung cancer link, despite clearly demonstrating cannabis smoke-induced cellular damage.

Furthermore, compounds found in cannabis have been shown to kill numerous cancer types including: lung cancer [9], breast and prostate [10], leukemia and lymphoma [11], glioma [12], skin cancer [13], and pheochromocytoma [14]. The effects of cannabinoids are complex and sometimes contradicting, often exhibiting biphasic responses. For example, in contrast to the tumor killing properties mentioned above, low doses of THC may stimulate the growth of lung cancer cells in vitro [15].

The genotoxic effects of partially oxidized hydrocarbons created by burning either cannabis or tobacco have been widely examined as the likely source of genetic changes that lead to the carcinogenic state [16]. As a result, the medical potential of cannabis has been obscured by the potential negative impact of using a smoked medicine [17]. Those who deny the validity of "medical marijuana," cite that marijuana smoke contains four fold more tars than does tobacco smoke [18]. Nevertheless, smoking is often the preferred route of intake by medical cannabis users because rapid action allows self-titration [19]. Are the biological consequences of smoking cannabis and tobacco similar?

Smoke from tobacco and cannabis contains many of the same carcinogens and tumor promoters [20,21]. However, cannabis and tobacco have additional pharmacological activities, both receptor-dependent and independent, that result in different biological endpoints. Polycyclic aromatic hydrocarbons found in smoke are pro-carcinogens that are converted to carcinogens by the enzymatic activity of the cytochrome P4501A1 oxidase protein (CYP1A1 gene product). Benzo [a] pyrene is converted to its carcinogenic metabolite diol epoxide, which binds to specific hyper-mutable nucleotide sequences in the K-ras oncogene and p53 tumor suppressor [22]. Recent work by Roth *et al*. demonstrates that THC treatment of murine hepatoma cells caused a dose dependent increase in CYP1A1 gene transcription, while at the same time directly inhibiting the enzymatic activity of the gene product [23]. Thus, despite potentially higher levels of polycyclic aromatic hydrocarbons found in cannabis smoke compared to tobacco smoke (dependent on what part of the plant is smoked), the THC present in cannabis smoke should exert a protective effect against pro-carcinogens that require activation. In contrast, nicotine activates some CYP1A1 activities, thus potentially increasing the carcinogenic effects of tobacco smoke [24].

It is worth noting that cytochrome P4501A1 oxidase has numerous substrates including biologically active lipid metabolites such as arachidonic acid, and eicosinoids [25]. These molecules are components of metabolic pathways that are interwoven with the synthesis and degradation of endocannabinoids such as arachidonylethanolamine (anandamide) [26]. Hence, the inhibition of cytochrome P4501A1 oxidase by THC is likely to have multiple biological effects such as possibly enhancing cannabinoid activities by decreasing their catabolism.

The need to better understand the biological consequences of tobacco compared to cannabis smoke has been underscored by recent studies that demonstrate a unique role for nicotine in the pathogenesis of lung cancer [27]. In order to appreciate potential biological differences between tobacco and cannabis smoke, the molecular basis of signal transduction must be considered with respect to the life and death of cells. Evolution has provided cells with biochemical feedback loops, checkpoints that monitor genetic integrity and the overall state of the cell. Under conditions of sufficient cellular damage, apoptotic cell death is induced [28]. While a variety of different biochemical states are consistent with a cell either living or dying, constant communication between a cell and its environment is critical for survival of the cell and ultimately the organism.

Cells communicate with each other via specific cell surface receptors. When bound with their appropriate ligand, the receptors initiate signaling cascades that alter cellular biochemistry [29]. THC found in cannabis [30] and nicotine found in tobacco [31] both have specific receptors by which their corresponding ligands modulate cellular functions. Interestingly, both cannabinoid [32] and nicotine receptors [27] are coupled to the AKT (PKB) signaling pathway. Activation of either receptor type can induce an anti-apoptotic state that prevents cell death. However, it is the context in which the AKT pathway is activated that determines whether an organism benefits or is harmed by this anti-apoptotic activity.

Nicotine receptors are widely distributed and are found in the epithelial cells lining respiratory passages. Cannabinoid receptors are also widely distributed, but have not been reported in respiratory epithelial cells. The differential expression of receptors may account for the apparent difference in carcinogenic activity that results from smoking tobacco compared to cannabis. Both types of smoke contain a complex mixture of compounds, some of which are carcinogenic. They both contain hot gasses and irritating particulate matter (tars). However, the anti-apoptotic response that results from the stimulation of the nicotine receptors, under mutagenic conditions, creates a worst-case scenario. The very cells that have accumulated sufficient genetic damage to normally initiate the apoptotic cascade are prevented from going down this suicidal path [33] even though it would be best for the organism as a whole. In contrast, when the AKT pathway is activated in the brain after head injury [34] or stroke, [35] cannabinoids protect against cell death to the organism's benefit. Likewise, nicotine can also activate the AKT pathway in the brain in a beneficial manner. For example, activation of the nicotine receptors, as is also true of cannabinoid receptors [36], can prevent the brain cell death that results from exposure to beta amyloid protein [37] as occurs in Alzheimer's disease.

The impact of receptor and downstream activation is complicated. Both nicotine and cannabinoids have been shown to effect angiogenesis in a receptor-mediated manner [13]. However, nicotine and tobacco have opposite effects on angiogenesis. Nicotine promotes neo-vacularization along with associated tumor growth, atheroma, up-regulation of VEGF, and cell migration [38]. In contrast, cannabinoids promote tumor regression in rodents and inhibit pro-angiogenic factors [39]. In fact, clinical trials to treat human glioma with THC have resulted in decreased levels of VEGF [40].

The signal transduction pathway described above represents one means by which the carcinogenic affects of tobacco are amplified in a contrasting manner to what occurs with cannabis. The immunological effects resulting from smoking tobacco or cannabis are also distinctive and result in opposite end-points. Again, the carcinogenic potential of smoke is increased by tobacco, whereas it is uniquely reduced by the specific immune regulatory activity of cannabinoids in cannabis smoke. The introduction of hot gaseous material containing both carcinogens and particulate material into the respiratory passages produces pro-inflammatory immune responses [41]. The inflammatory state is a double-edged sword that can serve to protect or kill an organism. A functional characteristic of the pro-inflammatory state is the production of free radicals [42]. These reactive chemical species are essential armaments in the body's defense against various pathogens, in particular against intracellular parasites and bacteria. Free radicals are thought to be contributing etiological agents behind a number of pathological states [43] including cardiovascular and neuro-degenerative diseases [44], cancers, and aging in general [45]. Endocannabinoids are specific immunological homeostatic modulators when acting on "peripheral" CB2 receptors [30]. Both endo- and exo-cannabinoids push the immune system towards the relatively anti-inflammatory Th2 cytokine profile [46]. Thus, cannabinoids inhaled in cannabis smoke physiologically reduce the potential amplification of carcinogens in smoke that results from biologically produced free radicals. This response is not induced by tobacco smoke.

In conclusion, while both tobacco and cannabis smoke have similar properties chemically, their pharmacological activities differ greatly. Components of cannabis smoke minimize some carcinogenic pathways whereas tobacco smoke enhances some. Both types of smoke contain carcinogens and particulate matter that promotes inflammatory immune responses that may enhance the carcinogenic effects of the smoke. However, cannabis typically down-regulates immunologically-generated free radical production by promoting a Th2 immune cytokine profile. Furthermore, THC inhibits the enzyme necessary to activate some of the carcinogens found in smoke. In contrast, tobacco smoke increases the likelihood of carcinogenesis by overcoming normal cellular checkpoint protective mechanisms through the activity of respiratory epithelial cell nicotine receptors. Cannabinoids receptors have not been reported in respiratory epithelial cells (in skin they prevent cancer), and hence the DNA damage checkpoint mechanism should remain intact after prolonged cannabis exposure. Furthermore, nicotine promotes tumor angiogenesis whereas cannabis inhibits it. It is possible that as the cannabis-consuming population ages, the long-term consequences of smoking cannabis may become more similar to what is observed with tobacco. However, current knowledge does not suggest that cannabis smoke will have a carcinogenic potential comparable to that resulting from exposure to tobacco smoke.

It should be noted that with the development of vaporizers, that use the respiratory route for the delivery of carcinogen-free cannabis vapors, the carcinogenic potential of smoked cannabis has been largely eliminated [47,48].

## Competing interests

The author(s) declare that they have no competing interests.
